# Lights and shadows on the role of rhG-CSF in cancer patients during the COVID-19 pandemic and future perspectives of research

**DOI:** 10.2217/imt-2021-0219

**Published:** 2021-09-29

**Authors:** Angioletta Lasagna, Marta Muzzana, Paolo Pedrazzoli

**Affiliations:** ^1^Medical Oncology Unit, Fondazione IRCCS Policlinico San Matteo, Pavia, 27100, Italy; ^2^Department of Internal Medicine and Medical Therapy, University of Pavia, Pavia, 27100, Italy

**Keywords:** cancer patients, COVID-19, cytokine storm, febrile neutropenia, proteomic profiling, recombinant human granulocyte-colony stimulating factors (rhG-CSFs)

## Introduction

Since the early phase of the COVID-19 pandemic, a principal international panel of experts from European Society for Medical Oncology (ESMO) [1] and Multinational Association of Supportive Care in Cancer (MASCC) [2], have drawn up recommendations for every aspect of the management of cancer patients.

In managing febrile neutropenia (FN), supportive care indications have driven to an expanded use of recombinant human granulocyte colony-stimulating factors (rhG-CSFs) to accelerate absolute neutrophil count recovery; in particular, they have also recommended its use in patients with intermediate risk (10–20%) and all patients with FN antecedents without risk factors [1,2].

G-CSF is a glycoprotein that stimulates granulopoiesis and leads to proliferation, maturation and mobilisation of neutrophils [3]. It is mainly expressed by myeloid cells, but most recent studies have reported its expression in fibroblasts, endothelial cells, bone marrow stromal cells, placenta, adult neural stem cells, B cells and cardiomyocytes [3]. Filgrastim, the first rhG-CSF approved by the US FDA in 1991, has renal- and neutrophil-mediated clearance pathways, while the serum concentrations of long-acting rhG-CSF are due to the neutrophil-mediated clearance [4]. Thus, in case of chemotherapy-induced neutropenia, the circulating concentrations of a single dose of long-acting rhG-CSF persist elevated until there is an increase in the absolute neutrophil counts (ANC), which increases the self-regulated clearance [4].

In this paper, the authors analyze the role of rhG-CSF. Eventhough, it was firstly emphasised, later it was considered with caution because of the risk of acute lung injury [5]. However, it is now proven to be effective because of its immunomodulatory action on the molecular pathophysiology of sepsis.

## What is rhG-CSF for & why is it used during the COVID-19 pandemic?

Before the COVID-19 pandemic, the prophylactic use of rhG-CSF [6] has been recommended by all guidelines when the overall risk of FN from the prescribed chemotherapy regimen is ≥20% [6]. Starting rhG-CSF treatment earlier during the COVID-19 pandemic may shorten hospital stay [7], and self-administration of rhG-CSF or the use of long-acting agents (e.g. pegfilgrastim) may reduce outpatient visits [8].

Some authors highlighted that the rhG-CSF administration may counteract with COVID-19-related lymphocytopenia and improve the outcome of the infection in patients without cancer [9,10]. Cheng *et al.* reported that rhG-CSF led to a sustained increase in lymphocyte, but they excluded patients with comorbidities such as cancer from the analysis, to minimise their potential adverse effects on the immune responses of the subjects [10].

We also found three case reports of agranulocytosis following COVID-19 infection responding to rhG-CSF without apparent adverse events [11–13]. Spencer described a case of pancytopenia in a 51-year-old man with natural killer (NK) cell large granular lymphocytic leukemia and mild COVID-19 treated with two doses of rhG-CSF on the first day and 5 days after the diagnosis of COVID-19 real time PCR (RT-PCR) positivity [11]. Lutfi reported a case of profound neutropenia 23 days from the beginning of COVID-19 infection, treated with a course of six doses of rhG-CSF from day 23 to 28 [12].

Finally, Hernandez described a case with FN successfully managed with a 7-day course of rhG-CSF 53 days from the beginning of COVID-19 infection [13]. The favorable use of rhG-CSF in these cases may depend on the time elapsed since the onset of acute COVID-19 when the cytokine activation phase has been resolved.

## Is rhG-CSF use dangerous in COVID-19?

Soon after the onset of the pandemic, some authors described the possibility of worsening of clinical conditions in cancer patients following the use of rhG-CSF during FN [14–17]. In particular, Nawar *et al.* illustrated the respiratory worsening of three cancer patients within 72 h after rhG-CSF administration [15]. These patients developed an increase in neutrophil-to-lymphocyte ratio (NLR), which suggests an imbalance in inflammatory response [15]. Taha *et al.* reported a clinical deterioration within 24 h after rhG-CSF administration in a patient with COVID-19 and leukopenia [16]. Malek suggested that concomitant COVID-19 infection in patients treated with rhG-CSF after hematopoietic cell transplantation (HCT) may lead to a worse clinical outcome [17].

rhG-CSF-induced neutropenia recovery may trigger off respiratory deterioration due to acute lung injury (ALI) or acute respiratory distress syndrome (ARDS) in neutropenic cancer patients with viral pneumonia [5]. ALI is due to the compartmentalisation of neutrophils in the lung with the formation of neutrophil extracellular traps (NETs), release of cytokines, reactive oxygen species and proteinases [18]. In COVID-19, excessive NETosis (a unique type of apoptosis caused by neutrophils), induced by epithelial and endothelial cells affected by the virus, is involved in the development of the ‘cytokine storm' with the elevation of plasma levels of chemokine such as IL-6, IL-7, IL-8, TNF-α and G-CSF [19].

Therefore, as remarked by Taha, rhG-CSFs therapy might worsen the overwhelming inflammatory reaction in COVID-19 and lead to worse outcomes [16].

Recently, Zhang *et al.* have reported a higher risk of hospitalisation, respiratory failure and death in cancer patients recovered from neutropenia through the administration of rhG-CSF [20]. In particular, among hospitalised patients, the administration of rhG-CSF was linked to an increased need for high levels of oxygen supplementation and death (HR: 3.56, 95% CI: 1.19–10.2, p-value = 0.024). This effect was predominantly observed among the patients showing a robust neutrophil response (HR: 7.78, 95% CI: 2.05–27.9, p-value: 0.004) compared to those with less robust levels of response to G-CSF (HR: 4.04, 95% CI: 0.80–16.7, p-value: 0.086) [20].

This paper has confirmed our conclusion in the early phase of pandemic [14–17] that the use of rhG-CSF could worsen the clinical conditions of the patients with cancer in case of concomitant COVID-19 infection [14].

## Future perspectives

The particular sequence of events, triggered by a hyperinflammatory mechanism, makes COVID-19 management particularly difficult and general rules often do not apply to COVID-19 patients. The complex interplay between cytokines and cellular response interaction with G-CSF metabolism as well as the use of rhG-CSF remains a dilemma in cancer patients with COVID-19 infection. The question remains as to whether it is better to force neutrophil count recovery or to run the risk of severe respiratory complications. The available data are not sufficient to confirm this.

Answering this question may be the object of both biological and clinical studies. Identifying the patients' risk by using analytical quick technologies with more sensitive biomarkers of cellular activation is an ideal field for precision medicine research. Targeting G-CSF might be a therapeutic option, and knowledge of the serum level of G-CSF in patients with sepsis might indicate whether its administration may benefit patients with low G-CSF level or harmful for those with a higher G-CSF level [21]. Moreover, the primary role of neutrophils associated with critical illness in COVID-19 has been highlighted by a recent literature [22]. Meizlish demonstrated, through the use of proteomic profiling and a machine learning prediction algorithm, that elevations in circulating markers of neutrophil activation precede the onset of critical illness. They have identified that a group of proteins are early transcribed in neutrophil development, then stored into cytoplasmic granules, and released from mature neutrophils. High levels of G-CSF may stimulate emergency granulopoiesis, and IL-8 drives neutrophil migration into the lungs. Here, neutrophils are activated and release RETN, LCN2 and HGF, as well as other proteins with antimicrobial and inflammatory functions. Testing of these proteins, by utilizing a more precise medicine approach, may help to distinguish earlier subjects with an increased risk of developing complications related to the administration of rhG-CSF. This strategy should integrate the standard procedures of COVID-19 diagnosis, in order to distinguish the patients with possible complications.

Another research goal is to study different ways of immune stimulation and G-CSF release following short-acting and long-acting rhG-CSF administration to establish which one has better safety profile.

## Conclusion

In this commentary, we would like to emphasise the importance of obtaining more clinical and preclinical data to understand the complexity of G-CSF and its interaction with the immune system in cancer patients with COVID-19 disease.

We recognize as a limitation of this commentary that most of the references are anecdotal case reports. However, we would like to underline that in neutropenic cancer patients with COVID-19 infection, the possible risks versus the benefits of the rhG-CSF administration should be weighed carefully, as rhG-CSF may lead to worsening clinical and respiratory status.

The balance between the potential increased use of rhG-CSF in patients with cancer receiving chemotherapy and the concerns raised by some of the studies quoted might be addressed by prospective trials by utilizing a more precision medicine approach.

Therefore, big data analysis on a large number of patients may be useful to correlate ANC, inflammatory marker trend and disease outcome.
